# A systematic review of adverse events following immunization during pregnancy and the newborn period

**DOI:** 10.1016/j.vaccine.2015.08.043

**Published:** 2015-09-26

**Authors:** T. Roice Fulton, Divya Narayanan, Jan Bonhoeffer, Justin R. Ortiz, Philipp Lambach, Saad B. Omer

**Affiliations:** a Departments of Global Health and Epidemiology, Rollins School of Public Health, Emory University, 1518 Clifton Road, Atlanta, GA 30322, USA; b University Children’s Hospital (UKBB), University of Basel, Spitalstrasse 33, 4056 Basel, Switzerland; c Brighton Collaboration Foundation, Spitalstrasse 33, 4056 Basel, Switzerland; d Initiative for Vaccine Research, World Health Organization, Avenue Appia 20, 1211 Geneva, Switzerland; e Department of Pediatrics, Emory University School of Medicine, 1648 Pierce Drive NE, Atlanta, GA 30307, USA; f Emory Vaccine Center, Emory University, 201 Dowman Drive, Atlanta, GA 30322, USA

**Keywords:** Adverse events, Pregnancy, Vaccine safety, Maternal immunization, Case definitionsm, AEFI

## Abstract

In 2013, the WHO Strategic Advisory Group of Experts on Immunization (SAGE) requested WHO to develop a process and a plan to move the maternal immunization agenda forward in support of an increased alignment of data safety evidence, public health needs, and regulatory processes. A key challenge identified was the continued need for harmonization of maternal adverse event following immunization (AEFI) research and surveillance efforts within developing and developed country contexts. We conducted a systematic review as a preliminary step in the development of standardized AEFI definitions for use in maternal and neonatal clinical trials, post-licensure surveillance, and other vaccine studies. We documented the current extent and nature of variability in AEFI definitions and adverse event reporting among 74 maternal immunization studies, which reported a total of 240 different types of adverse events. Forty-nine studies provided explicit AEFI case definitions describing 35 separate types of AEFIs. We identified variability in how AEFIs were determined to be present, in how AEFI definitions were applied, and in the ways that AEFIs were reported. Definitions for key maternal/neonatal AEFIs differed on four discrete attributes: overall level of detail, physiological and temporal boundaries and cut-offs, severity strata, and standards used. Our findings suggest that investigators may proactively address these inconsistencies through comprehensive and consistent reporting of AEFI definitions and outcomes in future publications. In addition, efforts to develop standardized AEFI definitions should generate definitions of sufficient detail and consistency of language to avoid the ambiguities we identified in reviewed articles, while remaining practically applicable given the constraints of low-resource contexts such as limited diagnostic capacity and high patient throughput.

## 1. Introduction

Since 1990, the world has experienced a dramatic decrease in early childhood mortality. In 2013, the global under-five mortality rate (U5MR) was 46 deaths per 1000 live births, nearly half the rate U5MR of 90 deaths per 1000 live births in 1990 [1]. However, the rate of this reduction in under-five mortality is still insufficient to reach the Millennium Development Goals’ target of a two-thirds reduction of 1990 mortality levels by the year 2015 [2,3].

Compared to under-five mortality, declines in newborn mortality have been much slower to materialize. As of 2012, nearly 40% of all under-five child deaths occur in the neonatal period, i.e., babies in their first 28 days of life [4]. Additionally, in developing countries, nearly half of all mothers and newborns fail to receive skilled care during and immediately after birth. The World Health Organization (WHO) estimates that up to two-thirds of newborn deaths can be prevented if known, effective health measures are provided at birth and during the first week of life [4].

A potential strategy to address this global health need is the immunization of pregnant women to prevent diseases in their newborn children. Trans-placental transfer of antibodies has been demonstrated in several studies, and may confer protection against influenza during a newborn’s first months of life [5]. This strategy is buoyed by the success of the global Maternal Neonatal Tetanus Elimination Initiative and recent vaccine studies demonstrating that immunization of pregnant women decreases newborn influenza [6,7]. However, there are challenges to introducing immunization programs in antenatal care in resource-poor settings, requiring careful consideration of existing regulatory processes and expansion of the evidence base to take into account local public health needs to inform maternal immunization programs and policy [8,9]. The WHO/PATH Maternal Influenza Immunization Project aims to address some of these challenges – specifically with respect to vaccine distribution, logistics, and potentially vaccine hesitancy and uptake – by promoting the integration of immunization into antenatal care platforms in low- and middle-income countries [10].

There are limitations to vaccine safety data in pregnant women as pregnant women are seldom included in clinical trials [11]. Most safety information comes from observational studies and analysis of post-licensure surveillance systems, such as those for influenza vaccines [12]. In 2014, the WHO Global Advisory Committee on Vaccine Safety (GACVS) reviewed inactivated influenza vaccine safety in pregnancy and found no safety signals [13], and three recent systematic reviews of influenza vaccine safety in pregnancy have also been reassuring [14–16]. Nevertheless, the absence of global standard definitions for maternal immunization adverse events hinders comparisons of safety data across studies and geographic regions. For these reasons, the WHO and Brighton Collaboration are developing standardized adverse event definitions and reporting practices for use in clinical trials in pregnant women and other post-licensure vaccine safety monitoring.

The objective of this systematic review is to determine the extent and nature of variability in AEFI definitions and adverse event reporting among maternal immunization studies. The review aims to characterize the heterogeneity of AEFI definitions and reporting methods, which will directly inform ongoing vaccine safety standardization efforts for the purposes of clinical trial design as well as vaccine pharmacovigilance after licensing. These efforts will enhance collection, reporting, and comparison of clinical and post-marketing surveillance safety data—advancing our collective understanding of vaccine safety in pregnancy, and contributing to the harmonization of vaccine pharmacovigilance.

## 2. Methods

### 2.1. Eligibility criteria and assessment

#### 2.1.1. Types of studies

We included randomized controlled trials and observational studies that define one or more AEFIs for the purpose of safety monitoring. We also included reviews of maternal immunization studies; reviews were thought to potentially contain abstracted information on AEFI definitions that may not have existed, either at all or at the same level of detail, in the source publications. Maternal immunization reviews were therefore included to ensure that this content was not overlooked. We did not include unpublished studies.

#### 2.1.2. Types of participants

Studies chosen for review included pregnant women of all ages. Studies that did not explicitly include pregnant women, either exclusively or as part of an at-risk demographic, were excluded.

#### 2.1.3. Types of interventions

Eligible interventions included all vaccines evaluated in pregnant women.

#### 2.1.4. Types of comparisons

We included studies making any relevant comparisons of vaccines against a control, such as placebo, unexposed or untreated group, or alternate vaccine formulation.

#### 2.1.5. Types of outcome measures

Acceptable outcome measures included intervention efficacy, effectiveness, or safety. Specifically, studies that did not evaluate vaccine safety as a primary outcome were included if maternal, childbirth, or neonatal safety or adverse event data were reported.

#### 2.1.6. Other selection criteria

Study setting had no impact on inclusion. We included studies conducted in any country or region, in rural, urban, or mixed contexts, and in any participant setting such as in-hospital or incommunity. Five studies published in languages other than English were considered for inclusion, but none were included in the final review due to lack of translation capacity. There was no constraint on date of publication.

### 2.2. Search strategy

**Fig. 1.**
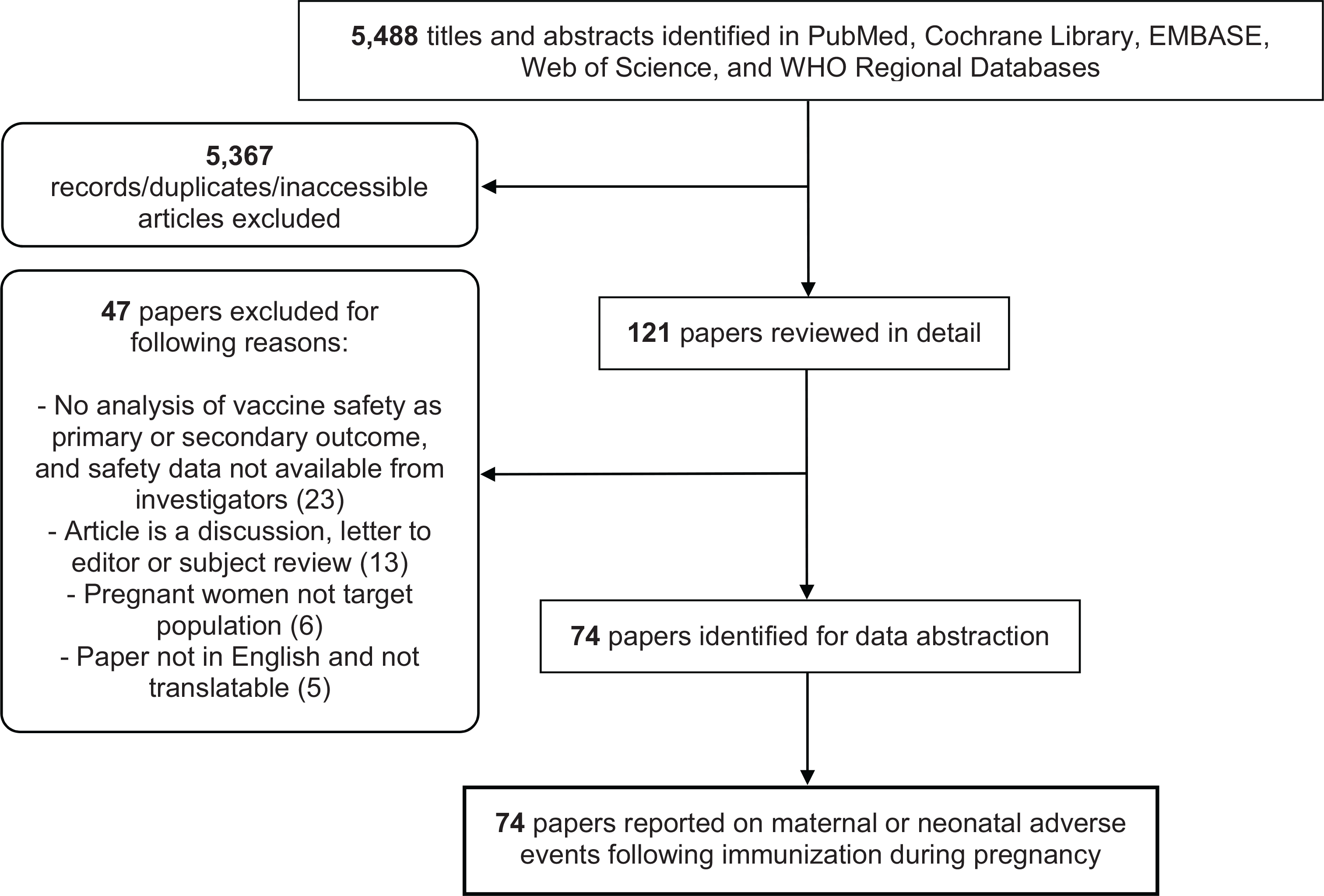
Systematic review workflow.

We conducted a comprehensive and systematic search of published literature potentially containing data on maternal and neonatal adverse events following maternal immunization (Fig. 1).

Sources included all published maternal immunization studies conducted to date (randomized controlled trials and observational studies), identified via searches of PubMed, EMBASE, Web of Science, and the Cochrane Database. The search strategy used for this review was derived from prior work by Bonhoeffer et al including a systematic review of vaccine safety data reporting [17]. Publications citing key papers that evaluated or attempted to establish immunization study reporting standards (e.g. Bonhoeffer et al. [17]) were also included in the search process. Supplementary Table 1 details our maternal AEFI search strategy on PubMed and EMBASE; Fig. 1 indicates the total number of results from all searches.

### 2.3. Screening and data extraction

The initial screening was conducted by one reviewer; a two-reviewer system was employed throughout the remainder of the review workflow. We imported search results into Endnote X5, and one reviewer (T.R.F.) screened titles and abstracts for eligibility. We discarded articles if their titles and abstracts clearly bore no relevance to this review. We retrieved full texts of eligible studies, and discarded inaccessible studies (six articles published prior to 2000, were inaccessible). Consensus and/or discussion with a second reviewer (D. N.) resolved uncertainty during the screening process with regard to inclusion/exclusion of studies. We recorded the rationale for study exclusion as part of the screening process.

The objective of our review was to determine the variance in AEFI definitions across all maternal immunization literature irrespective of study design, rigor, outcome, or potential bias. Therefore, a methodological study quality assessment (e.g., a Grading of Recommendations Assessment, Development and Evaluation (GRADE) analysis) was not required for the purposes of this review. Studies included in this review were neither assessed for, nor ranked on the basis of, limitations in design or possible bias.

We abstracted data from all research studies and publications meeting the inclusion criteria into an Excel workbook (Supplemental Table 2). We compiled additional data required to fully characterize AEFI definitions used into a set of queries. We contacted study investigators as necessary to request study protocols and/or to address omitted or incomplete data on adverse event definitions. We tracked communication with study investigators over the course of the review to ensure complete follow-up.

### 2.4. AEFI definitions and reported outcomes

We abstracted detailed information specific to AEFI case definitions into a comprehensive AEFI definition table (Supplemental Table 3). This table includes all additional unpublished information regarding AEFI definitions obtained via communication with study authors. Additionally, we abstracted detailed information specific to monitored and/or reported adverse event outcomes into a reported outcomes table (Supplemental Table 4). This table includes all reported or monitored maternal, fetal, and neonatal outcomes, irrespective of whether any study provided a case definition or classification code for the outcome.

Prior to performing analyses, we removed duplicate case definitions and definitions with minor (i.e., typographical and/or non-semantically significant, such as “fetal death” versus “death, foetal”) variations. However, we separately listed AEFIs that share similar definitions but are assigned different names. For example, “stillbirth” and “late fetal death” are separately listed despite both being defined as “fetal death occurring at or after 20 weeks gestation.” Similarly, we separately listed definitions for a single AEFI that share similar criteria (e.g., birth weight) but otherwise demonstrate semantically significant differences (for example, “birth after 20 weeks” versus “birth at or after 20 weeks”). Such AEFI names and definitions are considered “unique” for the purposes of our review.

### 2.5. Analysis

Data analysis was specifically tailored to provide the WHO and Brighton Collaboration a comprehensive overview of the heterogeneity among AEFI definitions and reporting methods in the maternal immunization literature

We performed the following analyses:

- Ranking of reported outcomes by frequency, using a series of generalized terms (e.g., miscarriage, preterm birth, stillbirth, etc.) to categorize each type of definition;
- Enumeration of studies providing AEFI definitions and/or information regarding classification systems used;
- Classification of AEFIs by individual affected (mother, fetus, or neonate);
- Ranking of AEFIs by frequency of definition and/or monitoring;
- Assessment of where in selected papers AEFIs are typically defined and/or reported on;
- Characterization of inconsistency in AEFI names and definitions, in terms of overall detail and terminology, thresholds and cut-offs, severity strata, and standards used; and
- Assessment of differences in studies’ definitions of what constitutes an adverse event.

## 3. Results

### 3.1. Study selection and characteristics

Out of a total of 5488 titles identified from electronic searches, 121 titles were selected as potentially relevant. All 121 papers were assessed for eligibility; following assessment, 47 papers were excluded with reason, and the remaining 74 papers were included in the review (Fig. 1). Of the 74 selected publications, ten were previously published reviews.

Half of the selected studies (37) focused on influenza vaccine, followed by yellow fever vaccines (5 studies), tetanus, diphtheria and acellular pertussis (Tdap) vaccine (4), and rubella vaccines (4) (Table 1). Other vaccines investigated included tetanus toxoid and vaccines against pertussis, diphtheria, varicella, group B streptococcus, hepatitis, human immunodeficiency virus (HIV), human papillomavirus (HPV), pneumococcal, cholera, cytomegalovirus, herpes simplex, meningococcal, polio, rabies, and respiratory syncytial virus. Five papers (of which three were reviews) examined vaccines against more than one pathogen.

The majority of studies were conducted in North America (35) or Europe (15) (Table 1). Of note, only two studies and two reviews were exclusively conducted in (or, in the case of reviews, provided AEFI definitions for studies exclusively conducted in) lower- or lower-middle-income countries [18–21]. Table 2 details these and other key characteristics of each study.

### 3.2. Reported adverse event outcomes

A total of 240 adverse events were reported among all selected publications (Supplemental Table 4). A significant proportion of these described typical pregnancy complications and a variety of congenital abnormalities. The specificity of adverse event reporting varied widely, with some events (e.g., “abnormal pregnancy,” “local reaction”) being vaguely defined or encompassing a range of adverse events. However, all reported AEFI definitions fell naturally within one of a series of AE categories specific to the AE target (mother, fetus, or neonate) (Table 4).

Of the 240 identified adverse events, 100 were specific to the mother, 21 to the fetus, and 119 to the neonate. Seventy-seven of the 119 neonate-specific adverse events were categorized as congenital malformations or abnormalities. Table 3 lists the ten adverse events most frequently reported.

### 3.3. Provision of AEFI definitions or classifications

Of the 74 selected studies, 49 provided one or more case definitions describing a total of 35 AEFIs. Across all studies, a total of 77 “unique” AEFI case definitions (excluding typographical or non-semantically significant variations) were found. Of the 77 unique case definitions, 11 definitions came from studies conducted in low- and lower-middle-income countries. The defined adverse events ranged in specificity, though most tended toward describing a single phenomenon (e.g., pre-eclampsia) rather than a group of related phenomena (e.g., “local reaction”). The most frequently defined AEFIs (stillbirth, miscarriage, preterm birth) were also the most often reported adverse events (Table 3). However, definitions were notably few in number for commonly expected or minor AEFIs such as site pain or fever. Table 4 lists all unique AEFI names and definitions, grouped by individual affected.

A substantial number of included studies (25) did not provide any AEFI definitions, but were included due to the reporting of AEFIs during pregnancy and the newborn period. Many of these studies cited the use of one of a number of adverse event classification systems in lieu of providing definitions (Supplemental Table 3); specific codes for some AEs were provided (Supplemental Table 4). Of note, most of these studies were retrospective analyses drawing data from registries and/or patient records.

### 3.4. Consistency of AEFI reporting

Of those studies providing AEFI definitions, most (particularly those examining maternal/fetal/neonatal AEFIs as the primary outcome) defined key AEFIs in Section 2 and subsequently reported on each in Section 3 (Supplemental Table 3). In total, 52 studies adhered to this reporting standard; of these studies, 38 reported on AEFIs as a primary outcome.

Studies that monitored or assessed a wide variety of adverse events, such as congenital abnormalities, typically specified a classification system used to detect or categorize adverse events (Supplemental Table 4). Classification systems in typical use included the International Classification of Diseases revision 9 (ICD-9) and the Medical Dictionary for Regulatory Activities (MedDRA). These studies often provided an exhaustive list of detected events in Section 3 without specific definitions.

### 3.5. Variability in adverse event definitions

Adverse event definitions were found to vary substantially between studies, as shown in Table 4. Specifically, definitions for key maternal/neonatal AEFIs differed on four attributes: overall level of detail, physiological and temporal boundaries and cut-offs, severity strata, and standards used.

#### 3.5.1. Level of detail

Definitions for key AEFIs varied widely in terms of terminology and level of detail. For example, Pasternak et al., 2012 defines miscarriage/spontaneous abortion as “abortion occurring between” certain weeks of gestation [22]; in contrast, Nishioka et al. [23] cite the WHO 1970 definition, which defines the same event as “any non-deliberate interruption of an intrauterine pregnancy before the 28th week of gestation in which the fetus is dead when expelled”. Other definitions use similar, but not identical, terminology, such as “pregnancy loss,” “fetal death,” and “intrauterine death.”

#### 3.5.2. Boundaries and cut-offs

**Fig. 2.**
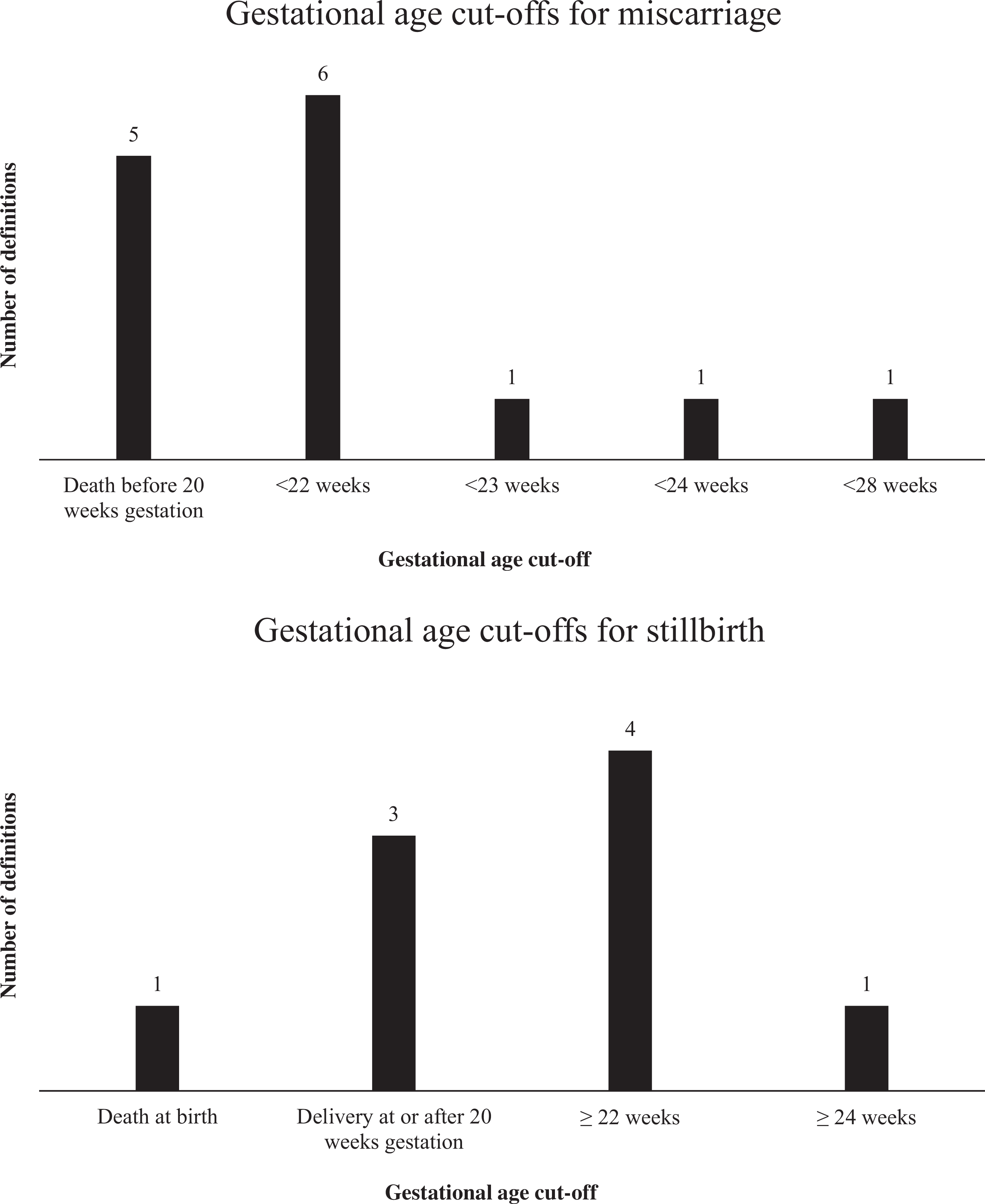
Gestational age cut-offs for (a) miscarriage/spontaneous abortion and (b) stillbirth.

Criteria for many defined AEFIs stipulate that a temporal or physiological threshold must be met or exceeded, such as fever being defined as temperature at or above 38 degrees Celsius. Here, study definitions revealed both stark and nuanced differences that may result in unexpected disagreements on AEFI classification across studies. This is best demonstrated by examining included studies’ gestational age cut-offs for miscarriage versus stillbirth. These cut-offs are defined using language that appears fairly homogenous (Table 4); however, upon close examination, substantial variations in gestational age thresholds become evident (Fig. 2a and b). One key issue is the fact that classification of miscarriage versus stillbirth will depend on whether the stated cut-off is inclusive or exclusive of (i.e., greater than versus greater than/equal to) the given gestational week. Such nuances in detail, both regarding these cut-offs as well as other factors (for example, some definitions also establish different criteria for when gestation actually begins) may introduce further uncertainty into the classification of fetal death.

In addition, some stillbirth and miscarriage definitions also included fetal mass criteria. This may raise the question of how to classify a fetal death should it (for example) meet gestational age, but not mass threshold, for a given definition.

#### 3.5.3. Severity strata

Eighteen papers in our review specified adverse event strata. For example, the Heikkinen et al., 2012 definition of stillbirth differentiated between “early fetal loss” as fetal death before 22 weeks gestation and “late fetal loss” as death between 22 and 28 weeks [24]. Other papers provided strata for preterm birth, low birth weight, and other AEFIs; these definitions varied on criteria, thresholds, and number of strata for a given AEFI (Supplemental Table 2).

#### 3.5.4. Standards used

Small for gestational age definitions exhibited variation in the use of fetal measurement standards. For example, Chambers et al. [36] and Cantu et al. [34] utilize Brenner and Lubchenco curves, respectively.

### 3.6. Defining an “Adverse Event”

In addition to the variability in definition provision and AEFI reporting, studies demonstrated substantial variation in what comprises an “adverse event”. Twenty-one studies offered explicit definitions of the term “adverse event” (Table 5). Among these definitions, six distinct variants of the term “adverse event” were described, e.g., “adverse obstetric event,” “adverse event of special interest,” etc. These definitions range broadly in specificity and, among definitions describing the same term, demonstrate little agreement even relative to that exhibited by more specific AEFI definitions. Table 5 summarizes all definitions provided for the term “adverse event” and variants used in selected studies.

## 4. Discussion

Our study revealed that three major forms of adverse variability currently exist among published maternal immunization studies, including variability in: AEFI definitions, AEFI reporting, and defining what constitutes an adverse event. Each of these can be mitigated by the future adoption of standardized definitions, which should be of sufficient detail and consistency of language to avoid ambiguity. Specifically, definitions should be consistent in defining key terms, diagnostic methodology, cut-offs, thresholds, severity strata, and physiological or other evaluative criteria. The high frequency with which we found certain AEFIs to be defined and reported (stillbirth, miscarriage, preterm birth, etc.) may also inform the priorities of standardization efforts.

Importantly, investigators may proactively work to eliminate between-study variability through clear and consistent reporting of AEFI definitions and outcomes in future publications. We observed that nearly all papers in our review that examined AEFIs as a primary study outcome provided definitions for each AEFI in Section 2. In addition, these investigators took care in Section 3 to report on each AEFI previously defined, whether or not they were detected. Although significant heterogeneity was still evident across the AEFI definitions provided in these papers, the consistency of this definition-and-reporting approach helped considerably to minimize confusion in reporting of outcomes, compared to studies that provided no definitions (or that only provided definitions parenthetically in Section 3).

We therefore recommend that case definitions for all monitored AEFIs in maternal immunization studies be explicitly provided in Section 2 of the study report or an online supplement, with the incidence of each defined adverse event reported on in Section 3 or an online supplement. If a classification system is used, it should be cited in Section 2. To further reduce ambiguity in comparing data, we recommend that classification codes be provided in the results tables for each detected adverse event. These recommendations are summarized in Table 6.

Special attention must be paid to AEFI definitions used in studies conducted in low- and low-middle-income countries. It may be reasoned that AEFI definitions need to take into account factors such as the relative lack of diagnostic capacity, high patient load, etc. that may be present in some low-resource settings, which may imply a preference for less-specific AEFI definitions compared to other contexts. An example would be a requirement to confirm preterm birth via early-pregnancy ultrasound in a setting with limited access to sophisticated diagnostic equipment.

Due to the very small number of studies conducted in low- and low-middle-income (LMIC) countries in our review, we are limited in our ability to assess meaningful differences in AEFI definitions in these contexts. Some of the unique AEFI definitions provided in our LMIC studies appear to reflect less interest in the specifics of an adverse event, and more interest in the sensitive detection of any abnormal event (e.g., “atypical infant behavior” and “severe acute maternal morbidity,” two catch-all definitions unique to LMIC studies). We do not, however, recommend that rigor in AEFI definitions should be relaxed for studies in LMIC contexts. Rather, considerations specific to low-resource countries should be given careful thought in the process of developing standard AEFI definitions.

Our study has additional limitations. We searched for articles via four major literature databases, but we did not hand-search individual journals or gray literature. Maternal immunization studies that reported no safety outcomes may nevertheless have monitored for AEFIs using established case definitions; however, these studies were not included in our review. Finally, six older (pre-2000) articles were excluded due to inability to access the full papers, and five non-English candidate articles were not included in the final review due to lack of translation capacity (Fig. 1).

Efforts to standardize maternal immunization AEFI definitions are ongoing, with active efforts underway by WHO, the Brighton Collaboration, and other contributors [25–27]. Our review has provided a more complete picture of the landscape of maternal AEFI definitions currently in use, to help inform how best to approach standardization. One of the principal standardization efforts, the Global Alignment of Immunization Safety Assessment in Pregnancy (GAIA), is a WHO/Brighton Collaboration joint project aiming to provide standards and tools to establish a globally shared understanding of outcomes and approaches to monitoring them, with specific focus on LMIC needs and requirements.

Future standardized AEFI definitions developed through such initiatives will help ensure consistency of data and facilitate data comparisons and pooling; however, they should be accompanied by consistency of reporting. We believe that this is achievable, and that improving adverse event reporting practices is a first step that should be taken without hesitation. In view of a swiftly expanding global maternal immunization agenda, we have no reason to delay.

## Supplementary Material

Supplementary Material 3

Supplementary Material 1

Supplementary Material 2

Supplementary Material 4

## Figures and Tables

**Table 1:** Summary characteristics of included studies.

	Number of papers

Selected publications by type	
Study type	
Retrospective cohort	30
Prospective cohort	24
Review	10
RCT	10
Cross-sectional	3
Case-control	2
Before/after	1
Selected publications by location	
Continent	
North America	35
Europe	15
South America	7
Asia	6
Africa	2
Australia	0
Multiple	9
Selected publications by vaccine	
Vaccine	
Influenza	37
Yellow fever	5
Tdap	4
Rubella	4
Varicella	2
Group B streptococcus	2
Hepatitis	2
HIV	2
Pneumococcal	2
HPV	1
Cholera	1
Cytomegalovirus	1
Herpes simplex	1
Meningococcal	1
Polio	1
Rabies	1
Respiratory syncytial virus	1
TT	1
Multiple	5

**Table 2:** Characteristics of selected studies.

Study authorand year [reference]	Continent	Study design	Type of vaccine	Primary study outcome	Safety-related outcome (if different from primary outcome)

Abzug, 2013 [28]	North America	Prospective cohort	Influenza	Safety and immunogenicity of H1N1 vaccine in HIV infected pregnant women	
Adedinsewo, 2013 [29]	North America	Retrospective cohort	Influenza	Maternal vaccination impact on prematurity and SGA	
Auffret, 2013 [30]	Europe	Prospective cohort	Influenza	Adverse event and vaccine safety of influenza vaccine in pregnant women	
Baker, 1988 [31]	North America	Prospective cohort	GBS	Antibody level in immunized pregnancies and in newborns	Maternal adverse effects
Baker, 2003 [32]	North America	RCT	GBS	Safety and immunogenicity in pregnant women	Maternal adverse effects
Bednarczyk, 2012^†^ [12]	Multiple	Review	Influenza	Safety of influenza immunization for fetus and neonate	
Black, 2004 [33]	North America	Retrospective cohort	Influenza	Impact of influenza vaccination on pregnant women and risk of illness and safety in newborns	
Cantu, 2013 [34]	North America	Retrospective cohort	Influenza	Association of influenza vaccination with increased risk of adverse pregnancy outcomes	
Cavalcanti, 2007 [35]	South America	Before/after	Yellow fever	Effect of yellow fever vaccine on newborn malformation rates	
Chambers, 2013 [36]	North America	Prospective cohort	Influenza	Risk and safety of H1N1 vaccines in women exposed during pregnancy	
Chavant, 2013 [37]	Europe	Prospective cohort	Influenza	Safety of a/H1N1 vaccination during pregnancy	
Christian, 2011 [38]	North America	Prospective cohort	Influenza	Inflammatory response to vaccination in pregnant women	
Conlin, 2013 [39]	North America	Retrospective cohort	Influenza	Safety of H1N1 vaccine in Pregnant US military women	
Cottin, 2013^†^ [40]	Multiple	Review	Yellow fever	Adverse events from yellow fever vaccine	
da Silva, 2011 [41]	South America	Prospective cohort	Rubella	Safety of Rubella vaccine during pregnancy	
Dana, 2009 [42]	Multiple	Prospective cohort	HPV	Safety of HPV vaccine during pregnancy-pregnancy outcomes and birth defects	
De Vries, 2014 [43]	Europe	Prospective cohort	Influenza	Adverse events of adjuvanted a/H1N1 vaccination during pregnancy	
Ergenoglu, 2012 [44]	Europe	Prospective cohort	Rubella	Safety of Rubella vaccine during pregnancy	
Harjulehto-Mervaala, 1994 [45]	Europe	Prospective cohort	OPV	Fetal development and perinatal outcome after OPV vaccination during pregnancy	
Hashim, 2012* [18]	Africa	Cross-sectional	Cholera	Birth outcomes between exposed and unexposed pregnancies	
Heikkinen, 2012 [24]	Multiple	Prospective cohort	Influenza	Influenza vaccine safety in pregnant women and neonates	
Huang, 2013 [46]	Asia	Prospective cohort	Rabies	Safety of post-exposure prophylaxis during pregnancy	
Kallen, 2012 [47]	Europe	Retrospective cohort (registry data)	Influenza	Pregnancy outcomes post H1N1 vaccination	
Kharbanda, 2012 [48]	North America	Prospective cohort	Influenza	Adverse effects from trivalent or monovalent influenza vaccination during pregnancy	
Kharbanda, 2013 [49]	North America	Retrospective cohort	Influenza	Adverse events between exposed and unexposed pregnant women, specifically, preterm and small for gestational age births	
Launay, 2012 [50]	Europe	Prospective cohort	Influenza	Consequences of maternal vaccination on pregnancy outcomes and maternal seroprotection at delivery	
Lehmann, 2003*^†^ [19]	Asia	Review	Pneumococcal	Pneumococcal vaccine safety review	
Lin, 2012 [51]	Asia	Retrospective cohort	Influenza	Adverse events after AdimFlu-S vaccination in pregnant women	
Lin, 2013 [52]	Asia	Prospective cohort	Influenza	Immune response of the three vaccine viral strains	Incidence of pre-specified adverse events and all serious/non-serious adverse events
Louik, 2013 [53]	North America	Prospective cohort	Influenza	Safety of H1N1 vaccine during pregnancy	
Ludvigsson, 2013 [54]	Europe	Retrospective cohort	Influenza	Adverse pregnancy outcomes from influenza H1N1 vaccination	
Mackenzie, 2012 [55]	Europe	Prospective cohort	Influenza	Adverse events and pregnancy outcome post H1N1 vaccination	
Makris, 2012^†^ [56]	Multiple	Review	Multiple	Safety of various maternal vaccines	
Moro, 2011 [57]	North America	Retrospective cohort	Influenza (monovalent)	Adverse events of monovalent influenza vaccination during pregnancy	
Moro, 2011 [58]	North America	Retrospective cohort	Influenza (trivalent)	Adverse events of influenza vaccine between exposed and unexposed pregnancies	
Moro, 2012^†^ [59]	Multiple	Review	Influenza	Safety of influenza vaccines on pregnant women and neonates with emphasis on a/H1N1 monovalent vaccine	
Moro, 2013 [60]	North America	Retrospective cohort	Influenza	Maternal and infant outcomes for vaccinated pregnant women	
Moro, 2014 [61]	North America	Retrospective cohort	Hepatitis	Vaccine maternal adverse effects	
Munoz, 2001 [7]	North America	RCT	Multiple	Safety and immunogenicity of PSV in pregnant women	
Munoz, 2003 [62]	North America	RCT	RSV	Safety and immunogenicity of RSV vaccine during pregnancy	
Munoz, 2005 [63]	North America	Retrospective cohort	Influenza	Safety of influenza vaccination during pregnancy	
Munoz, 2014 [64]	North America	RCT	Tdap	Safety of Tdap vaccine during pregnancy	Infant response to DTaP vaccine
Naleway, 2014^†^ [65]	North America	Review	Influenza	Safety of influenza vaccination during pregnancy	
Nishioka, 1998 [23]	South America	Case control	Yellow fever	Effect of yellow fever vaccination on spontaneous abortion	
Nordin, 2013 [66]	North America	Retrospective cohort	Influenza	Adverse Events after first trimester influenza vaccination	
Nordin, 2014 [67]	North America	Retrospective cohort	Influenza	Impact of influenza vaccine on preterm and SGA	
Omon, 2011 [68]	Europe	Prospective cohort	Influenza	Safety of non-adjuvanted H1N1 vaccine on pregnant women	
Oppermann, 2012 [69]	Europe	Prospective cohort	Influenza	H1N1 vaccine safety in pregnancy	
Orenstein, 2012*^†^ [20]	Africa	Review	Multiple	Develop estimates of maternal and neonatal background morbidity and mortality	
Pardon, 2011 [70]	South America	Prospective cohort	Rubella	Fetal adverse events after rubella vaccination in pregnant women	
Pass, 2009 [71]	North America	RCT	CMV	CMV infection	Vaccine adverse effects and birth outcomes
Pasternak, 2012 [22]	Europe	Retrospective cohort (registry data)	Influenza	a/H1N1 vaccination association with major birth defects, preterm birth and fetal growth restriction	
Pasternak, 2012 [72]	Europe	Retrospective cohort (registry data)	Influenza	Risk of fetal death and spontaneous abortion from vaccination against a/H1N1	
Pitisuttithum, 2011 [73]	Asia	RCT	HIV	Adverse events related and unrelated to pregnancy	
Quiambao, 2007* [21]	Asia	RCT	Pneumococcal	Immunogenicity and antibody transfer after pneumococcal vaccination	
Santosham, 2001 [74]	North America	RCT	Multiple	Safety and immunogenicity of Hib vaccines verses pneumococcal	
Sato, 2011 [75]	South America	Prospective cohort	Rubella	Fetal adverse events after rubella vaccination in pregnant women	Congenital rubella infection in newborns after exposure to vaccine
Shakib, 2013 [76]	North America	Retrospective cohort	Tdap	Safety of Tdap vaccine during pregnancy	
Sheffield, 2011 [77]	North America	Prospective cohort	Hepatitis	Feasibility and immunogenicity of an accelerated hepatitis B vaccine schedule in high-risk pregnant women	Maternal adverse effects
Sheffield, 2012 [78]	North America	Retrospective cohort	Influenza	First trimester influenza vaccination on neonatal outcomes	
Sheffield, 2013^†^ [79]	Multiple	Review	Multiple	Standardized vital signs and laboratory assessments during maternal vaccine trials	
Silveira, 1995 [80]	South America	Case control	TT	Safety outcomes of TT in newborns	
Suzano, 2006 [81]	South America	Retrospective cohort	Yellow fever	Safety of yellow fever during pregnancy	
Talbot, 2010 [82]	North America	Cross-sectional	Tdap	Safety of Tdap less than 2 year after previous tetanus vaccination	
Tavares, 2011 [83]	Europe	Prospective cohort	Influenza	Safety outcomes in exposed and unexposed women	
Tavares, 2013^†^ [84]	Multiple	Review	Herpes simplex	Risk of spontaneous abortion following HSV vaccination	
Thomas, 2012^†^ [85]	Multiple	Review	Yellow fever	Adverse events from yellow fever vaccine in vulnerable populations	
Toback, 2012 [86]	North America	Retrospective cohort	Influenza	Safety of LAIV during pregnancy	
Tsai, 2010 [87]	Europe	Retrospective cohort	Influenza	Pregnancy outcomes in exposed and unexposed women	
Wilson, 2008 [88]	North America	Retrospective cohort (registry data)	Varicella	Outcomes after inadvertent exposure to Varicella vaccine during pregnancy	
Wise, 2000 [89]	North America	Retrospective cohort	Varicella	Adverse events from varicella vaccine	
Wright, 1999 [90]	North America	RCT	HIV	Safety of rgp120 during pregnancy	
Zheteyeva, 2012 [91]	North America	Retrospective cohort	Tdap	Safety of Tdap in pregnant women	
Zheteyeva, 2013 [92]	North America	Retrospective cohort	Meningococcal	Safety of meningococcal vaccine in pregnancy	

* Study conducted in lower- or lower-middle-income country.

† Systematic review.

**Table 3:** Top ten adverse events most frequently reported in selected studies.

Adverse event	Number of times reported

Miscarriage/spontaneous abortion	31
Preterm birth	31
Stillbirth	25
Fever, maternal	19
Pre-eclampsia	14
Site pain	12
Low birth weight	12
Elective abortion	11
Respiratory distress	11
Small for gestational age	11

**Table 4:** List of unique adverse event definitions provided by selected studies. Only adverse events explicitly defined by study authors (i.e., those studies defining AEFIs beyond simply referring to the coding/classification scheme used) are shown. Definitions with an asterisk are those used in studies conducted in lower-middle-income countries (LMICs).

AEFI target (mother, neonate, fetus)	AEFI type	AEFI type, detail	AEFI definition, description, or classification reference used [references]

Mother	Local	Local reaction	Pain, redness, or swelling [82] Redness, swelling, induration or pain confirmed by comparison to a 2 cm circle (≥circle records as positive) [21]
	Systemic	Anaphylaxis	Physician assessment or Brighton definition [57,58] Sudden onset (<3d after vaccination) and rapid progression of symptoms involving multiple systems organ classes: dermatologic, cardiovascular, and respiratory (per Brighton collaboration definition) [40]
		Bell’s palsy	Brighton definition [57,58]
		Fever	“Feeling feverish” and/or temperature measured to be >100.4°F [82] Temperature ≥ 38 °C [51,52]
		Guillain-Barre syndrome	Physician assessment or Brighton definition [57,58]
		Influenza-like illness	Oral temperature > 37.8 °C with at least one influenza-like symptom (cough, sore throat, rhinorrhea, nasal obstruction) [50]
		Mild adverse event	Headache, fever, or myalgia [81]
	Pregnancy-related	Abnormal pregnancy	Ectopic pregnancy/spontaneous abortion/stillborn delivery [87]
		Maternal death	Death from direct or indirect obstetric causes during pregnancy or <42 days after pregnancy termination [20]*
		Normal pregnancy	Normal, live-born delivery [87]
		Pre-eclampsia	Pregnancies with an ICD-9-CM-coded diagnosis of preeclampsia (642.4×–642.7×) occurring during pregnancy [39]
		Pregnancy complications	Any or none, based on self-report [75]
		Preterm labor	Pregnancies with an ICD-9-CM-coded diagnosis of threatened premature labor (hereafter referred to as premature labor; 644.0×) or early (spontaneous) onset of delivery occurring during pregnancy with initial diagnosis of either premature labor or premature delivery at least 1 day after pandemic H1N1 or seasonal influenza immunization [39]
		Severe acute maternal morbidity	Direct or indirect obstetric complications that threaten the woman’s survival but do not lead to her death [20]*
Fetus	In utero	Elective abortion	Induced termination of a pregnancy due to personal choice or medical reasons prior to 20 weeks post-conception day (or 22 weeks of gestation) [83]
		Fetal death	Spontaneous abortion and stillbirth combined [42] Nonviable conceptus in pregnancies after more than 20 weeks amenorrhea [72]
		Induced abortion	Therapeutic or elective abortion [87]
		Intrauterine fetal death	Fetal death with unknown gestation time [57,58]
		Late fetal death	Fetal death occurring ≥20 w gestation [88]
		Miscarriage/spontaneous abortion	Abortion occurring between start of week 7 and end of week 22 of gestation [22] Any non-deliberate interruption of an intrauterine pregnancy before the 28th week of gestation (since the last menstrual period) in which the fetus is dead when expelled (WHO 1970)[23] Delivery between 14 w and 21 w + 6 days gestation [50] Delivery prior to 20 weeks [34] Fetal death occurring <20 weeks gestation [57–59,61,91,92] Intrauterine death of a fetus under 500 g or of gestational age under 22 weeks’ amenorrhea [37] Intrauterine death of fetus at <24 w gestation [83] Loss before 22 weeks gestation [24] Pregnancy loss before week 22 [43] Pregnancy termination within 20 weeks of conception [18]* Spontaneous loss of conceptus before 20 weeks amenorrhea [42] Spontaneous pregnancy loss at <20 gestational weeks [36] Termination of a pregnancy without human interference prior to 20 weeks post-conception day (or 22 weeks of gestation) [84] Loss of a fetus <500 g or prior to 22 weeks gestation [75]
		Stillbirth	Death at birth [20]* Delivery of a non-viable fetus at or after 20 weeks [34] Delivery of a non-viable fetus at or after 22 completed weeks [72] Fetal death occurring >20 weeks gestation [57,58] Fetal death of ≥500 g [57–60] Fetal demise ≥20 w gestation [91,92] Fetus born after 20 weeks gestation without pulse [18]* Intrauterine death of a fetus over 500 g or of gestational age more than 22 weeks’ amenorrhea [37] Intrauterine death of fetus at ≥24 w gestation [83] Loss of a fetus ≥500 g or at 22 weeks gestation or later [75] Pregnancy loss after week 22 [43]
Neonate	Congenital abnormality	Gross malformation	Physical defect present in baby at birth, including any abnormality visible on a naked baby (e.g. cleft lip or palate, Down syndrome, spina bifida, limb defects, etc.) [43]
		“Various”	Classified using CDC and Prevention Metropolitan Atlanta Congenital Defects Program guidelines. All structural–morphological, chromosomal or genetic anomalies were included in this definition, regardless of whether the fetus was delivered dead or alive, and included birth defects identified by prenatal ultrasound, amniocentesis or examination of the products of conception after elective or spontaneous abortion [83]
	Perinatal	Early neonatal death	Death of a liveborn infant within the first week of life [20]*
		Low Apgar score	Score < 7 at 5 min [50,54]
		Low birth weight	Birth weight < 2500 g [22,54,75,84] Birth weight < 2500 g measured by study team within 7 days of life or extracted from official birth records [20]*
		Postterm birth	Birth 42 weeks or greater [43]
		Preterm birth	Birth ≤ 36 completed weeks [54] Birth at <37 weeks gestation [75,84] Birth between 22 w and 36 w + 6 days gestation [50] Birth between 32 and 36 weeks [43] Clinical estimate of gestational age at birth of less than 37 completed weeks [29] Estimated gestational age <37 completed weeks as determined by ultrasound, last menstrual period, or validated exam within 7 days of life [20]* Live birth <37 weeks gestation [91,92]
		Small for gestational age	<10th percentile for sex and gestational age in live born infants using standard US growth charts for full and preterm infants (NCHS 2000 growth curves or Lubchenko) [36] <10th percentile of gestational age-specific birth weight within cohort [54,66] Brenner’s standard for fetal growth less than the 10th percentile [34] Children with measurements of weight, length, and head circumference <2 SD than reference values (calculated from full-term infant measurements of combined reference cohorts) [45] Lowest 10th percentile of birth weight for each gestational week [29] Measurements <2 SD from expected weight [47]
		Very low birth weight	Birth weight under 1500 g [42]
		Very preterm birth	Birth between 22 and 31 weeks [43] Birth less than 32 weeks gestation [42]
	Postnatal	Atypical infant behavior	Deviation from normal feeding, crying, defecating, urinating, sleeping, and growing behavior as defined by mother [18]*
		Infant death	Live births dying later in infancy [18]*
		Late neonatal death	Death of a liveborn infant between 1 and 4 weeks of life [20]*
		Recurring illness	Illness lasting more than two weeks or occurring twice or more often [18]*

**Table 5:** List of definitions of the term “adverse event” and variations, as detailed in selected studies.

Term	Definition [reference]

Adverse event	Any undesired, noxious or pathological change in participants as indicated by physical signs, symptoms, and/or laboratory changes that occurred following administration of one of the vaccines, whether or not considered vaccine-related (includes intercurrent illnesses or injuries and unexpected exacerbations of pre-existing conditions) [73] Any untoward medical occurrence in a patient or clinical investigation subject administered a pharmaceutical product regardless of its causal relationship to the study treatment. An AE can therefore be “any unfavorable and unintended sign(s), symptoms(s) or condition temporally related with the use of the investigational product” [77]
Adverse obstetric event	New, prespecified, medically attended pregnancy-related comorbidities or pregnancy complications [48]
Medically significant adverse event	Requiring two or more visits to a physician for the same condition or that resulted in hospitalization or an ER visit [73]
Adverse event of special interest	Any event considered as worthy of closer follow-up as described in recommendations for the Pharmacovigilance Plan following the administration of H1N1 pandemic vaccines [83]
Medically-attended adverse event	Event leading to an otherwise unscheduled visit to or from medical personnel for any reason, including visits to an accident and emergency department [83]
Neonatal adverse event	Visits with prespecified ICD-9 codes that occur from birth through 30 days old [49]
Serious adverse event	Defined per FDA guidelines [71] Any untoward medical occurrence that: resulted in death, was life-threatening, required hospitalization or prolongation of existing hospitalization, resulted in disability/incapacity, or was a congenital anomaly/birth defect in the child of a study participant [78] Reports containing information that the AE resulted in death, hospitalization, prolongation of hospitalization, life-threatening illness, persistent or significant disability, or congenital anomalies. Definition modified to exclude reports on hospitalizations for delivery unless they required prolonged stay in a hospital due to delivery complications or postpartum conditions [90] AEs resulting in or prolonging hospital admission, is life-threatening, fatal or resulting in significant or persisting disability [55] AEs including death, hospitalization, and cause for hospitalization and permanently disabling conditions [64] Any adverse experience any time after vaccination that resulted in any of the following outcomes: death, a life-threatening adverse experience, inpatient hospitalization or prolongation of existing hospitalization or a persistent or significant disability/incapacity, a congenital anomaly, or an important medical event that, based upon medical judgment, may have required medical or surgical intervention to prevent one of those outcomes [82] Deaths, life-threatening events, hospitalizations, persistent or significant disabilities, congenital anomalies, and other events of medical importance (FDA regulations) [89] Any event leading to hospitalization, prolonging hospitalization, entailing permanent handicap or disability, and life-threatening or fatal condition (per French public health code) [37] VAERS Report classified as serious if resulting in death, life-threatening illness, hospitalization or prolongation of hospitalization, permanent disability, or congenital anomaly [57–60]

**Table 6:** Summary of recommendations for future efforts in standardization of AEFI definitions.

Recommendation 1. Case definitions should be of sufficient detail and consistency of language to avoid ambiguity with respect to:	- Cut-offs/thresholds defining related AEs - Severity strata - Physiological or other evaluative criteria - Diagnostic methodology - Definition of key terms (e.g., gestational age as weeks of amenorrhea or as weeks post-conception)
Recommendation 2. Effort should be made to encourage the following qualities:	- Consistent reporting in Section 2 of AE definitions used or specific classification schemes/codes (e.g., ICD-9 codes) in all studies - Consistent reporting in results of all AEs defined
Recommendation 3. Explore the use of frequency analysis to inform standardization efforts where multiple case definitions for a given AE are available	
Recommendation 4. Prioritize appropriate standardization for future studies over “back-compatibility” with definitions in previous studies, given the substantial variability in existing definitions	
