# Supplementary material for: A systematic review of adverse events following immunization during pregnancy and the newborn period
Source: Vaccine. Author manuscript; Available in PMC 2021 Jul 20. (PMC8290429; doi:10.1016/j.vaccine.2015.08.043)
Supplement: Supplementary Material 4 [file NIHMS1714969-supplement-Supplementary_Material_4.docx]

**Supplemental Table S1** Maternal immunization literature search strategy for PubMed and EMBASE (descending order; results for PubMed shown).

|  | **Term** | **Hits** |
| --- | --- | --- |
| **#18** | **#5 AND #11 AND #17 NOT "animals"[MeSH Terms:noexp]** | **4219** |
| **#17** | #12 OR #13 OR #14 OR #15 OR #16 | 2701526 |
| **#16** | Search (adverse event or adverse effect or side effect or undesirable effect or injurious effect or adverse event following immunization or aefi or risk management or risk factor or risk factor analysis[Text Word]) | 1353122 |
| **#15** | Search safety[MeSH Terms] | 53042 |
| **#14** | Search risk management[MeSH Terms] | 201703 |
| **#13** | Search risk factors[MeSH Terms] | 550598 |
| **#12** | Search adverse effects[MeSH Subheading] | 1690689 |
| **#11** | #6 OR #7 OR #8 OR #9 OR #10 | 910202 |
| **#10** | Search (matern* or mother* or prenat*[Text Word]) | 453701 |
| **#9** | Search maternal exposure[MeSH Terms] | 5111 |
| **#8** | Search prenatal care[MeSH Terms] | 19986 |
| **#7** | Search mothers[MeSH Terms] | 25496 |
| **#6** | Search pregnancy[MeSH Terms] | 704478 |
| **#5** | #1 OR #2 OR #3 OR #4 | 610523 |
| **#4** | Search (vaccin* or immuni* or inoculat*[Text Word]) | 600688 |
| **#3** | Search immunization[MeSH Terms] | 132799 |
| **#2** | Search vaccination[MeSH Terms] | 56158 |
| **#1** | Search vaccines[MeSH Terms] | 169435 |
